# Hypercalcemia of Malignancy Revealing a Parathyroid Carcinoma with Hepatic Metastasis: A Case Report and Literature Review

**DOI:** 10.1155/2020/8883413

**Published:** 2020-11-13

**Authors:** Yassine Mellagui, Rachid Jabi, Inass Haouli, Christine Kora, Jamal Ouachaou, Mohammed Aabdi, Houssam Bkiyar, Mohammed Bouziane, Brahim Housni

**Affiliations:** ^1^Department of Anesthesia-Intensive Care Unit, Faculty of Medicine and Pharmacy of Oujda, Mohammed VI Hospital Center, Mohammed I University, Morocco; ^2^Department of General Surgery, Faculty of Medicine and Pharmacy of Oujda, Mohammed VI Hospital Center, Mohammed I University, Morocco; ^3^Department of Nephrology and Renal Transplantation, Faculty of Medicine and Pharmacy of Oujda, Mohammed VI Hospital Center, Mohammed I University, Morocco; ^4^Department of Radiology, Faculty of Medicine and Pharmacy of Oujda, Mohammed VI Hospital Center, Mohammed I University, Morocco

## Abstract

Parathyroid carcinoma is a very rare malignant tumor of the parathyroid gland. This cancer poses a great diagnostic and therapeutic difficulty due to its rarity and the absence of a characteristic clinical and paraclinical picture. The diagnosis is histological but is not always easy. Surgery remains the only curative treatment, and cervical radiotherapy can be discussed. Good prognostic factors are complete monobloc tumor resection, and bad prognostic factors are the presence of lymph node metastases at diagnosis, distant metastases, and nonsecreting carcinomas.

## 1. Introduction

Parathyroid carcinoma is a rare malignant tumor that represents less than 0.005% of all cancers and 1% of the tumors of the parathyroid gland; its diagnosis is difficult because this cancer has no clinical or biological specificity compared to parathyroid adenoma. It should be suspected in the presence of severe primary hyperparathyroidism associated most often with palpable cervical mass [1, 2].

## 2. Case Presentation

A 54-year-old male patient with no significant medical history consulted for diffuse bone pain with impaired general condition. The clinical examination revealed the pain of all the bony segments without other associated signs. The biological assessment had shown severe hypercalcemia with a calcemia corrected to 229 mg/l (5.72 mmol/l), a normal phosphoremia at 0.87 mmol/l, hyperparathyroidism with a serum PTH level very high at 2315 pg/ml (27 times normal), renal failure with plasma creatinine at 29 mg/l (257 *μ*mol/l), and plasma urea at 1.03 g/l (17.1 mmol l) with preserved diuresis (0.7 ml/kg/h). No other abnormalities were found.

Faced with the threat of hypercalcemia, the first phase of rehydration based on 0.9% saline (4 l per day) was recommended, but without improvement in the calcium level, hence the need to start dialysis (hemodialysis). The patient had benefited from 2 hemodialysis sessions with improved calcemia (173 mg/l) and renal function (creatinine at 14 mg/l).

**Figure 1:**
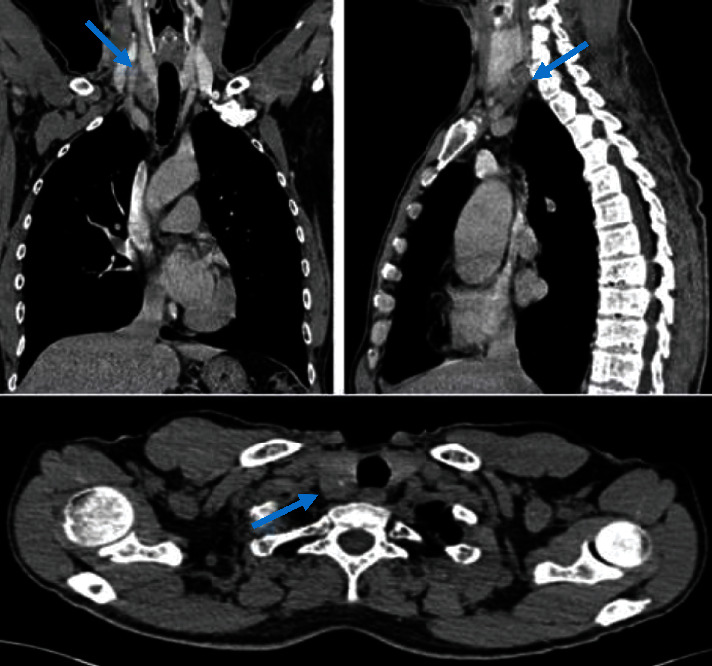
Cervicothoracic tomodensitometry: heterogeneous hypodense nodule in the posteroinferior pole of the right thyroid lobe.

**Figure 2:**
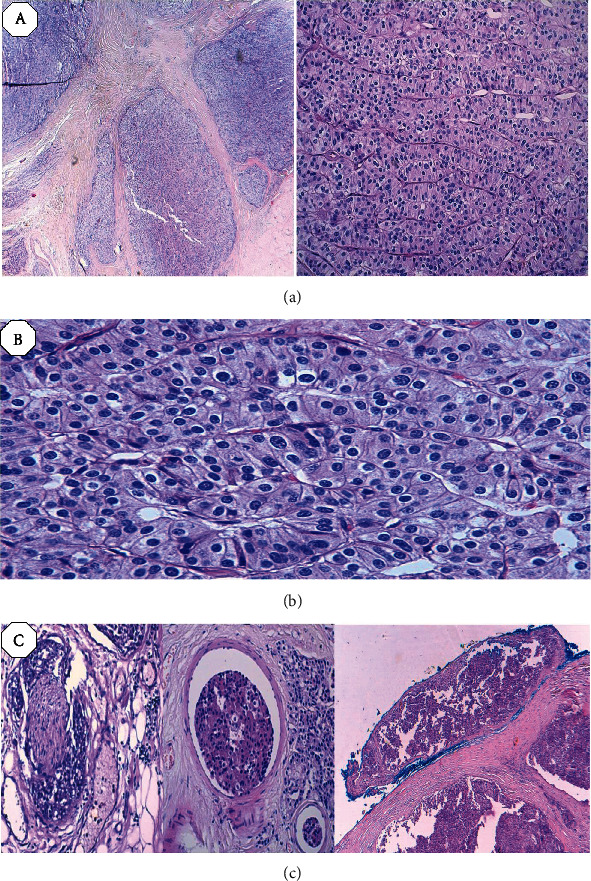
Photomicrographs of the histopathological examination showing tumor proliferation made of uniform cells arranged in lobules, trabeculae, and clusters that are separated by dense fibrous bands (a). Neoplastic cells are roughly polygonal, with an atypical nucleus, prominent nucleoli, and abundant cytoplasm, and mitotic figures are frequent (b). Vascular and capsular invasions were observed inside the tumor and in the surrounding tissues. The tumor also displays evidence of capsular invasion (c).

Standard skeletal X-rays showed diffuse bone demineralization. The cervical ultrasound showed a heterogeneous hypoechoic right tissue mass with intimate contact with the homolateral thyroid lobe. The cervicothoracic tomodensitometry (TDM) concluded that there was a heterogeneous hypodense nodule in the posteroinferior pole of the right thyroid lobe (Figure 1). At the end of this assessment, the diagnosis of a parathyroid adenoma was mentioned and surgery was indicated. The surgery consisted of a right lower parathyroidectomy removing the nodule after releasing it from the thyroid lobe. The final anatomopathological examination concluded with a parathyroid carcinoma with total resection R0 (Figure 2).

**Figure 3:**
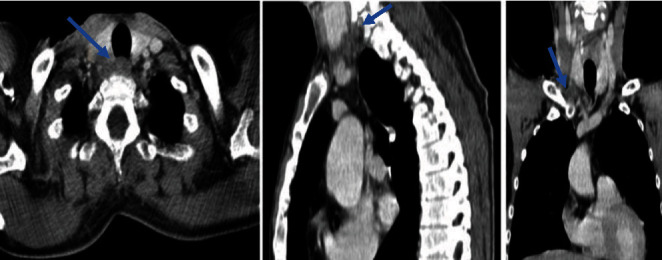
Postoperative cervicothoracic tomodensitometry: tissue infiltrate concerning exeresis lodge.

**Figure 4:**
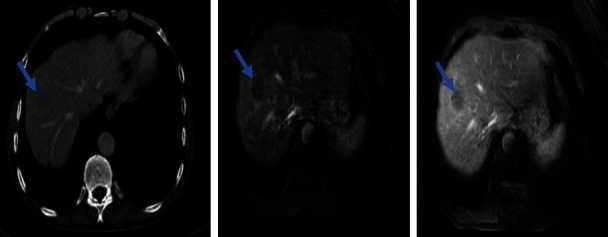
Suspected hepatic segment VIII lesion. The same lesion is in hyposignal T1 and hypersignal T2.

The postoperative follow-ups were marked by the persistence of hypercalcemia between 133 and 151 mg/l and serum PTH level values between 2300 and 2884 pg/ml requiring the performance of another cervico-thoraco-abdomino-pelvic scan which objectified a tissue infiltrate concerning exeresis lodge (Figure 3) with a secondary hepatic lesion. An MRI was requested for a more precise study of the number and location of the liver lesions which revealed a suspicious lesion of segment VIII of the liver (Figure 4). Tc-99m-sestamibi scintigraphy did not show any fixation anomalies in favor of residue or other parathyroid lesions.

The therapeutic approach was to treat the hepatic lesion by radiofrequency with improved postoperative hypercalcemia, and dialysis sessions were stopped. Following the patient's refusal and after a multidisciplinary discussion, no adjuvant treatment was instituted. Three months later, the patient returned to the emergency room with malignant hypercalcemia and died following the failure of all medical measures to reduce calcium levels.

## 3. Discussion

Parathyroid carcinoma is a very rare malignant tumor of the parathyroid gland; it is endocrine neoplasia that affects less than 1% of patients with primary hyperparathyroidism. This cancer should be suspected in front of a picture of severe clinical and biological hyperparathyroidism, with high rates of calcemia and serum parathyroid hormone, whether or not associated with cervical mass [3].

In many studies, parathyroid carcinoma affects women more than men, with a sex ratio of 5/1 and an average age of 45-50. The first case of parathyroid carcinoma was described by Quervain in 1909, and in 1938, Armstrong published the first case of parathyroid carcinoma revealed by primary hyperparathyroidism [4].

The series by Lee et al. which includes 224 parathyroid carcinomas between 1988 and 2003 showed that the incidence of this tumor had gone from 3.58 to 5.73/10,000,000, with a frequency of postoperative discovery of the order of 78.6% [5].

Parathyroid gland cancer poses a great diagnostic and therapeutic challenge due to its rarity and the absence of specific clinical and paraclinical signs, which leads to error towards benign primary hyperparathyroidism [6]. Its etiopathogenesis is poorly understood, but it appears that some genetic and environmental factors interact in a very complex way. Neck irradiation, especially at a young age, increases the risk of parathyroid neoplasia [7].

The clinical manifestations of parathyroid carcinoma are those found in cases of severe hyperparathyroidism such as osteoporosis, renal failure, and hypercalcemia. Unusual symptoms can be found such as fatigue, weight loss, abdominal pain, nausea, and vomiting [8]. The clinical examination may find a palpable cervical mass sometimes adhering to the thyroid gland or adjacent structures (muscle, soft tissues, and recurrent laryngeal nerve) [9]. 15-30% of patients have lymph node metastases, and 1/3 of patients have distant metastases usually in the lungs, liver, and bones [10].

In addition to these clinical features, the severity of primary biological hyperparathyroidism should be considered very suspect. Several studies find that 65 to 75% of patients have a calcemia greater than 3.50 mmol/l. Similarly, the significant rise in blood parathyroid hormone levels is suspect. This rate has been reported on average to 10 times normal in cases of carcinoma and 2.6 times normal in patients with primary benign hyperparathyroidism [6].

Regarding imaging, parathyroid cancer appears in ultrasound as a hypoechogenic lobulate lesion with irregular limits [7]. Scintigraphy with Tc-99m-sestamibi is very useful for tumor localization and characterizes abnormal or ectopic parathyroid tissue. But it does not provide information about the benign or malignant nature of the tumor. However, it could also be useful in the diagnosis and localization of metastases of parathyroid carcinomas [11]. The TDM/MRI has a better sensitivity than the scintigraphy of Tc-99m-sestamibi, but with a similar specificity [12].

Like many endocrine neoplasias, the distinction between benign and malignant parathyroid tumors is difficult. In 1973, Schantz and Castleman defined a set of histological criteria for the diagnosis of parathyroid carcinoma based on an analysis of 70 affected patients: the presence of parenchymal mitoses, trabeculated parenchyma including thick fibrous band, and capsular or vascular invasion [13]. However, the most specific signs of malignancy are capsular, vascular, and/or lymphatic invasion [14].

Surgical resection is the basic treatment of parathyroid carcinoma; it must include an exeresis of the parathyroid tumor and homolateral thyroid lobe and a recess of the pretracheal and recurrent lymph node chains [12]. Postoperative radiation therapy does not have a major place in the treatment of parathyroid carcinoma, because this cancer is radioresistant [15], but radiation therapy can be beneficial in terms of lower recidivism [16].

Patients who benefit from a complete monobloc tumor resection may have high survival rates which reach up to 90% at 5 years and 67% at 10 years. The factors of poor prognosis are the presence of lymph node metastases at the time of diagnosis, distant metastases, and nonsecreting carcinomas [2].

## 4. Conclusion

Parathyroid carcinoma is a rare hypersecreting malignant tumor with a difficult diagnosis, whose treatment is primarily surgical. The prognosis depends mainly on the possibility of performing complete surgical exeresis. Complementary postoperative radiation therapy can be beneficial in terms of lower recidivism rates. The severity of this condition is due to severe hypercalcemia, which increases mortality, and the risk of recurrence and distant metastases which require prolonged surveillance.
